# A Critical Appraisal of the Definition of Sarcopenia in Patients with Non-Alcoholic Fatty Liver Disease: Pitfall of Adjusted Muscle Mass by Body Weight

**DOI:** 10.3390/life10100218

**Published:** 2020-09-23

**Authors:** Huiyul Park, Dae Won Jun, Hoon-ki Park, Kye-Yeung Park, Minki Kim, Hwan-Sik Hwang

**Affiliations:** 1 Department of Family Medicine, Hanyang University College of Medicine, Seoul 04763, Korea; bliss153@hanmail.net (H.P.); kyeyeung@naver.com (K.-Y.P.); a7921@naver.com (M.K.); fmhwang@hanyang.ac.kr (H.-S.H.); 2 Department of Internal Medicine, Hanyang University College of Medicine, Seoul 04763, Korea

**Keywords:** NAFLD, sarcopenia, relative muscle mass

## Abstract

Traditionally, sarcopenia has defined as amount of absolute muscle mass adjusted by height in the elderly people. However, relative muscle mass adjusted by weight has been used extensively in most non-alcoholic fatty liver disease (NAFLD) studies. Here, we attempted to investigate the pitfall of adjusted muscle mass by weight to evaluate association between sarcopenia and NAFLD. Adult subjects (n = 1343) who underwent a health check-up were finally included for analysis. The weight-adjusted skeletal muscle mass index (wSMI) and height-adjusted SMI (hSMI) calculated by dividing the total appendicular skeletal muscle (ASM) by weight or the square of height, respectively. Prevalence of sarcopenia defined by wSMI in the NAFLD group was significantly higher than in the control group (1.3% vs. 8.8%, *p* < 0.001). However, there was no difference in the prevalence of sarcopenia defined by hSMI between the control and NAFLD groups (2.0% vs. 0.8%, *p* = 0.055). Since body weight was the most potent independent risk factor for NAFLD in multivariable logistic regression analysis, abnormal rates (<−1 SD) of almost all parameters increased in the NAFLD population, after weight adjustment. However, abnormal rates of non-metabolic parameter did not increase in NAFLD, after height adjustment. Only metabolic parameters showed relationship with NAFLD, after height adjustment. As NAFLD is highly associated with body weight, careful attention should be given in the case of studying the relationship of NAFLD with sarcopenia adjusted by body weight.

## 1. Introduction

Sarcopenia is characterized by an age-related decrease in muscle mass and muscle strength. The prevalence of sarcopenia is 10–40% in people aged 65 or older [1]. Recently, it has been determined that sarcopenia is more closely related to metabolic diseases, beyond the decline and quality of life in older patients [2,3,4]. Non-alcoholic fatty liver disease (NAFLD) and sarcopenia have common characteristics such as insulin resistance and chronic inflammation [1,5]. Muscle is a representative target organ of insulin, and various myokines secreted from muscle are also known to influence NAFLD development [1,5].

The Sarcopenia Working Group recommends calculation of the skeletal muscle index (SMI) as the appendicular skeletal muscle (ASM) divided by the square of the height [6]. However, SMI adjusted by body weight or body mass index (BMI), instead of height, is more extensively used in studies to investigate the association between metabolic diseases and sarcopenia.

There have been four meta-analyses of the association between NAFLD and sarcopenia [7,8,9]. All meta-analyses demonstrated that the prevalence of sarcopenia was higher in the NAFLD group than in the control group. Moreover, sarcopenia itself increases the risk of accelerated disease progression [5,7,8,9].

However, 17 of the 19 studies in the meta-analysis of the relationship between NAFLD and sarcopenia used the SMI adjusted by weight (wSMI) for the sarcopenia diagnosis. All 17 of these studies showed a higher prevalence of sarcopenia in the NAFLD group [10].

Among the 19 studies, only two (Shen et al. [11] and Zhai et al. [12]) adjusted for sarcopenia by height (using hSMI). In these two studies, the prevalence of sarcopenia in the NAFLD group was rather low, and not significantly different from that of the control group. Peng et al. also reported the same result. Characteristics of the sarcopenia group differed markedly according to the two different adjustment methods, and the prevalence of sarcopenia showed an association with NAFLD only in the case of adjustment by weight [13].

The adjustment of muscle mass by height is estimating the absolute muscle mass. Whereas, the adjustment of muscle mass by body weight is estimating the relative muscle mass, which is also mathematically equivalent to estimating the amount of body fat. As NAFLD is highly associated with body weight, careful interpretation should be given when studying the relationship of NAFLD with any parameters adjusted by body weight.

In this study, we will compare the prevalence of sarcopenia according to adjustment methods and evaluate the pitfalls of muscle mass adjustment by either weight or height in association with sarcopenia and NAFLD.

## 2. Materials and Methods

### 2.1. Study Design

This study was designed as a cross-sectional study. The medical records of health check-up visitors at Hanyang International University Hospital were collected retrospectively for analysis. The Institutional Review Board (IRB) of Hanyang University Medical Center approved this study protocol (IRB No. HYUH 2019-10-013-002). The protocol was also registered at the Clinical Research Information Service (https://cris.nih.go.kr/cris). The informed consent was waived, because suspected harm to participants was minimal or none.

### 2.2. Inclusion and Exclusion Criteria

**Figure 1:**
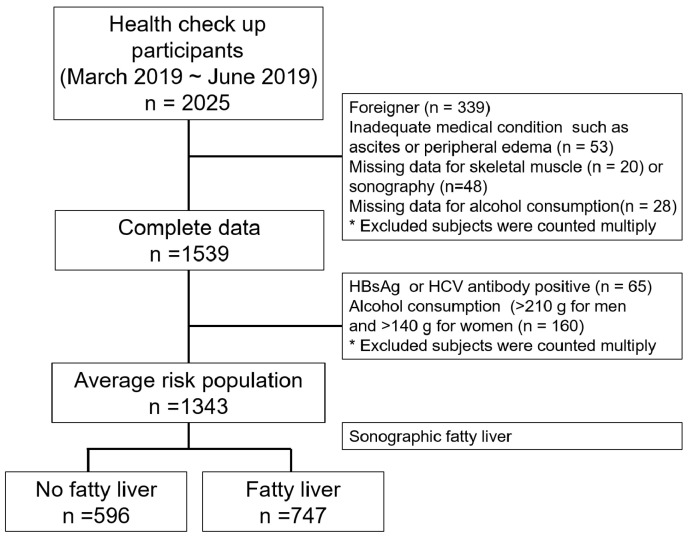
Study flow diagram.

In this study, a total of 2025 adult subjects who underwent a health check-up at the healthcare service at the Hanyang University Hospital between March 2019 and June 2019 were initially recruited into the study. We collected the data, excluding foreigners (n = 339), subjects with peripheral edema or ascites due to various medical conditions (n = 53), and subjects with missing data for baseline physical measurements including skeletal muscle mass (n = 20), abdominal sonography (n = 48), or alcohol consumption (n = 28). We further excluded the high risk liver disease population, meaning subjects who had positive serologic markers of hepatitis B virus (HBV) or hepatitis C virus (HCV) or who self-reported as having chronic hepatitis B infection (n = 65) or subjects whose weekly alcohol consumption was greater than 210 g for men or greater than 140 g for women (n = 160). Finally, 1343 subjects were included in this study (Figure 1).

### 2.3. Clinical Variables and Laboratory Evaluations

Personal medical and medication history, smoking history, exercise, and alcohol consumption were collected through a self-reported survey. Body weight and height were measured, and BMI was calculated as weight in kilograms (kg) divided by the square of the height in meters (m^2^). Waist circumference (WC) was measured at the narrowest point between the iliac crest and the lower rib margin. Blood pressure was measured at rest in a sitting position.

The following serum biochemical variables were measured using conventional methods: aspartate aminotransferase (AST), alanine aminotransferase (ALT), c-glutamyl transferase (GGT), alkaline phosphatase (ALP), total bilirubin (T. Bil), direct bilirubin (D. Bil), total cholesterol, triglyceride (Tg), high-density lipoprotein (HDL) cholesterol and low-density lipoprotein (LDL) cholesterol, fasting glucose, and platelet count (PLT).

### 2.4. Definition of Sarcopenia

Total skeletal muscle (TSM), appendicular skeletal muscle (ASM), and fat were measured using bioelectrical impedance analysis (BIA; InBody 720 body composition analysis). The weight-adjusted skeletal muscle index (wSMI), height-adjusted SMI (hSMI), and BMI-adjusted SMI (bSMI) were calculated by dividing the total ASM, which is the sum of skeletal muscle in the bilateral upper and lower four limbs (kg), by body weight (kg) in percent (total ASM/body weight × 100%), by the square of height (total ASM/height^2^), and by BMI (total ASM/BMI), respectively. The cutoff values for sarcopenia were defined for the hSMI as <6.58 kg/m^2^ for men and <4.59 kg/m^2^ for women, for the wSMI as <29.1% for men and <23.0% for women, and for the bSMI as <0.789 for men and <0.521 for women [14,15].

### 2.5. Definition of NAFLD

The non-alcoholic fatty liver was defined by results of abdominal ultrasonography. Significant alcohol intake was defined as men who consumed more than 210 g of alcohol per week and women who consumed more than 140 g of alcohol. Subjects with a history of HBV and HCV hepatitis or other endocrine disorders, those with clinically significant liver or renal function abnormalities, and on medications that may result in fatty liver, such as steroids, amiodarone, or herbal medicines were excluded. Subjects with NAFLD include all of the subjects with non-alcoholic fatty liver (NAFL) and non-alcoholic steatohepatitis (NASH) without consideration of the hepatic fibrosis or hepatitis. The degree of fatty liver was graded as normal, mild, moderate, or severe fatty liver based on the degree of fat infiltration [16]. The degree of fat infiltration was evaluated by the following ultrasound parameters, such as liver echotexture, liver attenuation, and visualization of intrahepatic vessel borders or diaphragm.

### 2.6. Statistical Analysis

Participants were divided into 4 groups on the basis of the severity of fatty liver. Continuous and categorical variables are presented as mean (SD) and number (percent), respectively. For calculating *p* for the trend of continuous variables among the 4 groups, Analysis of variance (ANOVA) was used. For calculating *p*-value of continuous variables between subjects without NAFLD and with NAFLD, T-test was used. Chi-square test was used for categorical variables. Fisher’s Exact test was used for categorical variables for which 20% or more of the cells had an expected frequency less than five. The in-concordance rate in Table A2 was calculated by dividing the difference between subjects with sarcopenia by wSMI and hSMI by the total number of subgroup in percent.

The relationship between the component of SMI such as ASM, body weight and height, and NAFLD was assessed using logistic regression analysis at Table 2. Multiple models adjusted for confounding factors, which have been known to having a strong relationship with fatty liver [17] and the component of SMI, were constructed as follows: Model 1 was adjusted for age; Model 2 was further adjusted for height; Model 3 was further adjusted for weight. Model 4 was additionally adjusted for WC, fasting glucose, Tg, AST, and ALT. The results are reported as odds ratios (OR) with 95% confidence intervals (CI). For all analyses, *p*-values less than 0.05 were considered as statistically significant. Statistical analyses were performed using SPSS, version 20.0 for Windows (SPSS Inc., Chicago, IL, USA).

## 3. Results

### 3.1. Baseline Characteristics

From all health check-up participants, 1343 subjects were finally included for analysis, after 682 subjects with risk factors for liver disease were excluded (Figure 1). The baseline characteristics, demographic data, and sarcopenia prevalence of the 1343 study subjects according to presence of NAFLD or the hepatic steatosis status are presented in Table 1. The mean age of the subjects was 46.8 years, and the proportion of males was 57.6%. Age, body weight, BMI, cholesterol, and fasting blood glucose were higher in subjects with NAFLD than those without NAFLD (Table 1). Not only total fat mass (19.26 kg vs. 14.29 kg, *p* < 0.001), but also total skeletal mass (TSM) (28.36 vs. 25.14, *p* < 0.001) and appendicular skeletal mass (ASM) (21.40 vs. 18.92, *p* < 0.001) were higher in the subjects with NAFLD than the control group. However, there was no difference in the amount of exercise between the groups with and without NAFLD (data are not shown).

### 3.2. Prevalence of Sarcopenia According to Adjustment Methods and the Concordance Rate

**Figure 2:**
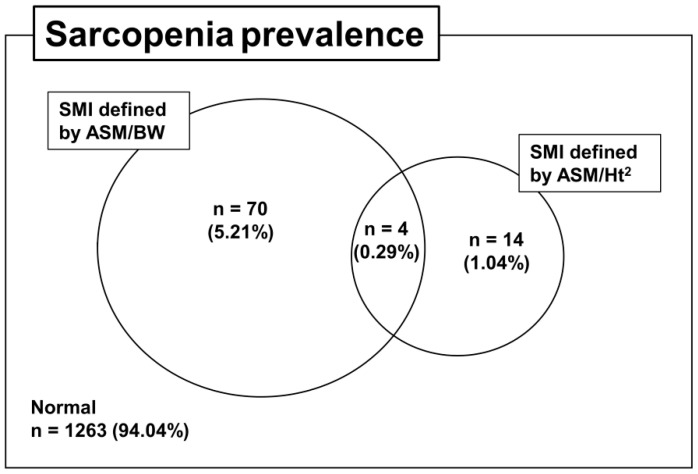
Venn diagram of the prevalence of sarcopenia according to different skeletal muscle index (SMI) definitions.

**Figure A1:**
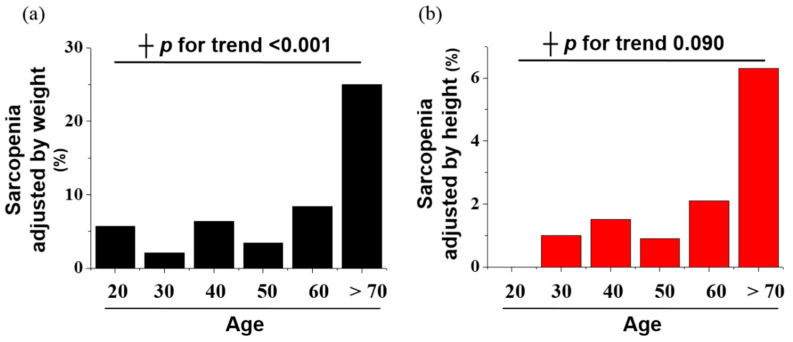
The graph of sarcopenia prevalence adjusted by body weight (**a**) and by height (**b**) versus age. The *p* for trend was calculated by using ┼ Fisher’s Exact test.

The prevalence of sarcopenia was assessed by using wSMI and hSMI in Figure 2 and according to age in Figure A1. As the age increased, the prevalence of sarcopenia defined by hSMI increased significantly (*p* for trend: <0.001), but the prevalence of sarcopenia defined by wSMI did not increase significantly (*p* = 0.09). The prevalence of sarcopenia according to wSMI and hSMI was 5.5% (74/1343) and 1.33% (18/1343), respectively, in the whole population. In particular, only 0.29% (4/1343) of the participants satisfied the criteria for sarcopenia according to both wSMI and hSMI at the same time (Figure 2).

### 3.3. Associations between NAFLD and Sarcopenia According to Two Types of Adjustment Methods

**Figure 3:**
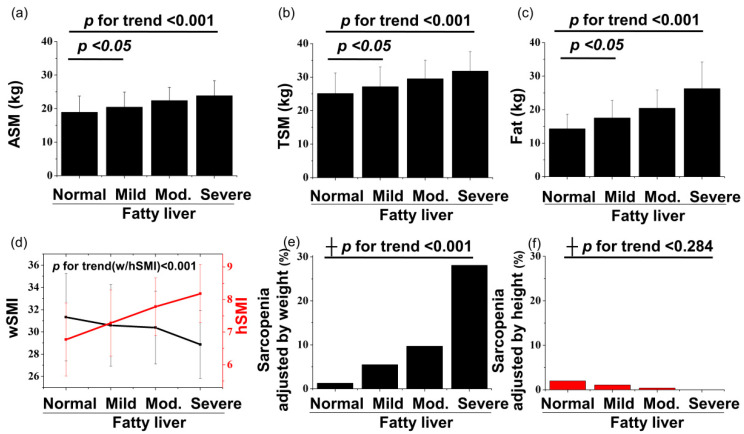
Graphs of ASM (**a**), TSM (**b**), and fat (**c**) versus the severity of fatty liver. Graphs of skeletal muscle index (**d**) and sarcopenia prevalence (**e** and **f**) versus the severity of fatty liver, according to different adjusting methods. The *p* for trend of continuous and categorical variables was calculated using ANOVA and ┼ Fisher’s Exact test, respectively. *p* values between the normal and mild fatty liver severity groups for ASM, TSM, and fat were calculated using *t*-test.

The prevalence of sarcopenia showed opposite directional tendencies according to the different methods of adjusting muscle mass (Figure 3). The prevalence of sarcopenia defined by wSMI was higher in subjects with NAFLD, 8.8%, than in the control group, 1.3% (*p* < 0.001), and the prevalence increased as the severity of fatty liver increased (Table 1). The prevalence of sarcopenia defined by bSMI also showed similar pattern with that of wSMI (Table 1 and Table A1). Since body weight is potent risk factor for NAFLD, the result by the BMI adjustment method containing body weight also shows similar features to that by the body weight adjustment method. Conversely, the prevalence of sarcopenia defined by hSMI was lower in subjects with NAFLD, 0.8%, than in the control group, 2.0% (*p* = 0.055), and the prevalence of sarcopenia adjusted by height showed a negative correlation with severity of NAFLD in Table 1. Moreover, absolute ASM and TSM were higher in subjects with NAFLD than in the control group, and their values also increased as the severity of fatty liver increased (Figure 3a–c). These pattern was also observed in age-stratified analysis with a 20-year interval (Table A1).

### 3.4. Pitfalls and Risk of the Weight Adjustment Method in NAFLD

**Figure 4:**
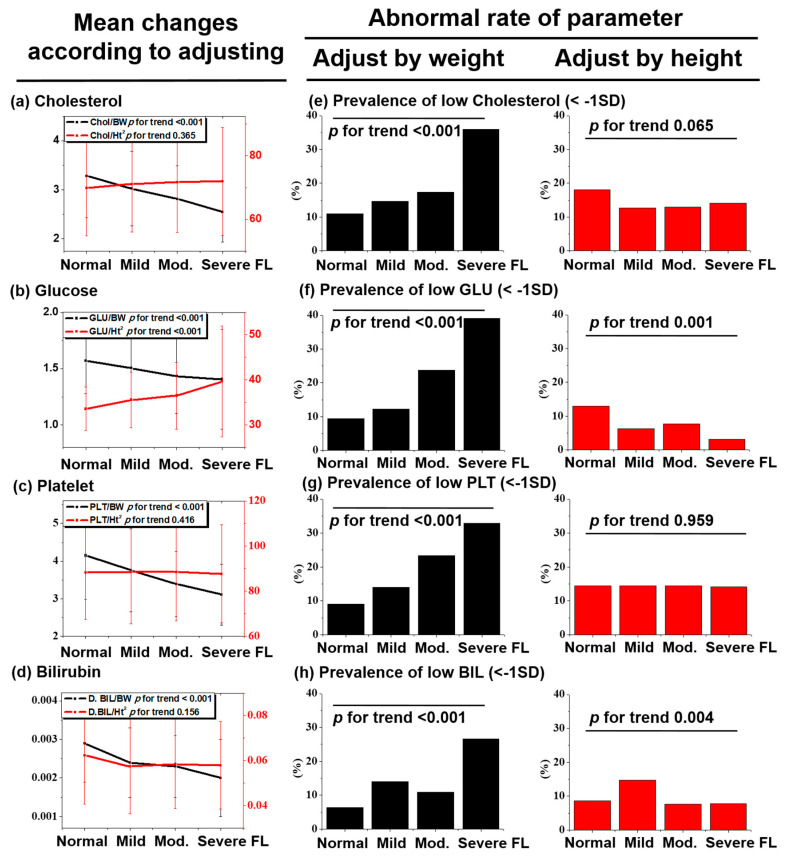
Graph of the variables, which are (**a**) total cholesterol, (**b**) glucose, (**c**) platelet, and (**d**) direct bilirubin, adjusted by weight or height versus the severity of fatty liver. Comparison of the prevalence of metabolic abnormality according to the adjustment method (**e**–**h**). Metabolic abnormality is defined as a blood level of the given parameter being lower than −1 SD. The *p* for trend of continuous and categorical variables was calculated using ANOVA and Pearson Chi-square test, respectively.

In order to investigate the pitfalls of the weight adjustment method on sarcopenia in patients with NAFLD, we examined the association of ‘metabolic parameters’ that are well known risk factors for NAFLD and ‘non-metabolic parameters’ that are not related to NAFLD (e.g., platelet and bilirubin, and so forth) with NAFLD adjusted by weight or height (Figure 4). Not only ‘metabolic parameters’ (Figure 4a,b, black line, *p* < 0.001) but also ‘non-metabolic parameters’ (platelet and bilirubin) (Figure 4c,d, black line, *p* < 0.001) came to have an association with NAFLD, after weight adjustment. In addition, the abnormal rate (<−1 SD) of metabolic and non-metabolic parameters (Figure 4e–h, back box, *p* < 0.001) increased significantly, as the severity of hepatic steatosis increased after weight adjusting. However, there was no association of non-metabolic parameters (Figure 4c,d, red line) and abnormal rate of the non-metabolic parameters (Figure 4g,h, red box) with NAFLD after height adjustment.

### 3.5. Risk Factors for NAFLD Determined with Multivariate Analysis

**Figure A2:**
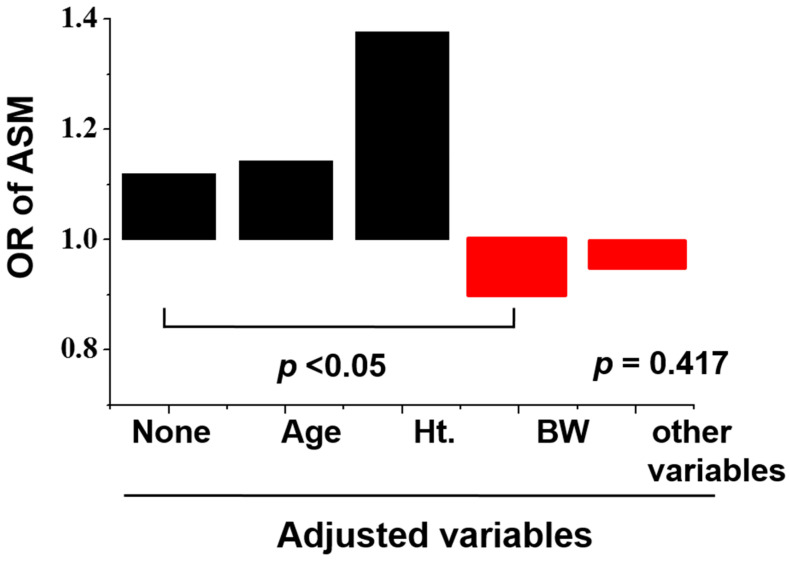
The graph of OR of ASM versus adjusted variables used in logistic regression. The analysis was carried out with corrections in order of age, Ht, BW, and other variables, which has been known for having a strong relationship with NAFLD in previous study, cumulatively.

We tried to find an independent risk factor for NAFLD. We calculated the odds ratio (OR) of ASM for NAFLD comparing participants with and without NAFLD by using a multi-model logistic regression in Table 2. ASM is rather an independent risk factor for fatty liver after adjusting age and height. However, when body weight was adjusted for Model 3, the OR of ASM was 0.899 (*p* = 0.022) and the ASM showed a negative independent risk factor for NAFLD (Figure A2). When metabolic parameters were adjusted additionally at Model 4, statistical significance of ASM for NAFLD disappeared.

## 4. Discussion

In this study, the absolute amounts of ASM and TSM in subjects with NAFLD was higher than those in the control group. When hSMI was applied for the diagnosis of sarcopenia, the prevalence of sarcopenia in subjects with NAFLD was lower than that in the control group. However, when wSMI was applied, the prevalence of sarcopenia in subjects with NAFLD was higher than that in the control group.

Guidelines suggest the use of ASM or ASM divided by the square of height, or hSMI, as an indicator of the relative muscle index [6]. However, wSMI or bSMI, which contain body weight as a component, were used more widely in most studies to investigate the relationship between NAFLD and sarcopenia. Thus far, nineteen studies have been performed to examine the relationship between sarcopenia and NAFLD. Among them, only two studies used the hSMI method, and only in those two studies was sarcopenia found to not be linked to NAFLD [10]. Shen et al. [11] and Zhai et al. [12] showed either no association between sarcopenia and NAFLD (OR = 1) or a reduced risk for NAFLD (OR = 0.48). Zhai et al. explained that, because muscular function and subject age were not considered for analyses, different results were observed [12]. However, Peng et al.’s research [13], where subjects were recruited with limitations of muscle function and age, also revealed differences according to the adjustment method. Therefore, we think that the SMI adjustment method is the most important cause of these observable differences.

NAFLD is a metabolic disease influenced by body weight. Therefore, if a target variable is adjusted by weight for analysis, weight can act as a confounder. In the study by Tsai KS et al. wSMI showed a negative correlation with the metabolic parameters of waist circumference, body mass index, and amount of fat. In contrast, hSMI was positively correlated with the same metabolic parameters [18]. In addition, hSMI was more highly correlated with the five muscular function indicators (hand grip, gait speed, leg endurance, cardiopulmonary endurance, and flexibility). Tsai KS et al. proposed hSMI as the more suitable criterion when defining sarcopenia because hSMI correlated with grip strength better than wSMI, and wSMI showed a high negative correlation with percentage of fat [18]. This is also supported by the Asian Working Group for Sarcopenia (AWGS) consensus [19].

In addition to the correlation with muscular function, the independency with obesity is considered as an important factor for skeletal muscle index [18,20]. In order to know the effect of body weight or obesity, BMI-stratified prevalence of sarcopenia by two different adjustment method was evaluated in Table A2. As BMI is containing body weight as a component, similar pattern prevalence of sarcopenia according to status of BMI defined by two different adjustment methods showed different tendency was also observed. Moreover, the higher in-concordance rate (16.57% in obese group; 10.0% in underweight group) was observed, compared to that in normal weight group (1.3%). This results mean that difference in sarcopenia prevalence resulted from two different adjustment method increases, as the abnormality of body weight increases. We think that hSMI, which is less affected by weight than wSMI, is a more suitable criterion based on this point.

Peng et al. also recently questioned the usage of wSMI in assessing the risk of sarcopenia for those with NAFLD [13]. In order to confirm this, they evaluated the prevalence and risk of sarcopenia for NAFLD according to adjustment methods. Peng et al.’s findings also showed that the risk of sarcopenia for subjects with NAFLD increased when wSMI was used for the diagnosis of sarcopenia. On the contrary, the risk decreased when hSMI was used. In addition, they proposed that weight could act as a confounder, because body weight includes body fat, which is a key component of NAFLD and one of the potential causes resulting in these differences.

Our study has several limitations. First, there was no consideration of muscular function and age in defining sarcopenia. However, the purpose of our study was not focused on studying the relationship between sarcopenia and sarcopenia-related diseases or the functional loss, but rather the relationship between the adjustment method of muscle and NAFLD. In the future, it is necessary to examine the relationship between muscular function and relative skeletal muscle according to the adjustment method. Second, the bioimpedance analysis (BIA) device, known to be less accurate than Dual energy X-ray absorptiometry (DEXA) in measuring muscle mass, was used. BIA analysis can be used to measure total muscle mass relatively easily but may be inaccurate when measuring subjects with edema. However, this study was performed on healthy subjects at check-up, except for people with edema due to heart, kidney, and liver disease. Third, the research design is cross-sectional, which cannot establish a causal relationship. To compensate for this, further longitudinal studies are needed.

Due to logical problems, careful attention should be paid when interpreting studies that used a weight-adjusted method to examine the correlation between sarcopenia and metabolic disease.

## Figures and Tables

**Table 1:** Baseline characteristics, demographic data, and sarcopenia prevalence of study subjects according to presence of NAFLD or hepatic steatosis status.

Health Check-Up Participants (N = 1343)	NAFLD (−) n = 596	NAFLD (+) n = 747	Hepatic Steatosis Status of Subjects With NAFLD			*p*-Value *	*p* for Trend
			Mild FL n = 434	Moderate FL n = 249	Severe FL n = 64		
Age (year)	44.13 (13.0)	48.94 (10.764)	48.76 (11.2)	49.59 (10.0)	46.80 (10.0)	<0.001	<0.001
Sex Male (n [%])	238 (31.9)	509 (68.1)	260 (59.9)	197 (79.1)	52 (81.3)	<0.001	<0.001
BMI (kg/m^2^)	21.64 (2.56)	24.88 (3.07)	23.86 (2.66)	25.71 (2.60)	28.54 (3.59)	<0.001	<0.001
Body weight (kg)	59.89 (10.66)	70.27 (11.77)	66.48 (10.32)	73.62 (10.26)	82.83 (13.87)	<0.001	<0.001
Fat (kg)	14.29 (4.37)	19.26 (6.11)	17.55 (5.20)	20.43 (5.45)	26.28 (7.97)	<0.001	<0.001
ASM (kg)	18.92 (4.83)	21.40 (4.47)	20.45 (4.50)	22.41 (3.96)	23.89 (4.45)	<0.001	<0.001
TSM (kg)	25.14 (6.12)	28.36 (5.93)	27.18 (5.83)	29.53 (5.57)	31.80 (5.83)	<0.001	<0.001
Height (cm)	165.87 (8.68)	167.70 (8.26)	166.61 (8.25)	168.99 (7.79)	170.05 (9.01)	<0.001	<0.001
WC (cm)	73.01 (7.56)	82.73 (8.62)	79.55 (7.60)	85.55 (6.75)	93.34 (9.48)	<0.001	<0.001
Platelet count (×10^3^/µL)	240.45 (47.43)	244.23 (51.89)	243 k (52.64)	244 k (50.86)	250 k (51.08)	0.329	0.414
Fasting glucose (mg/dL)	91.60 (9.91)	101.08 (17.13)	98.09 (14.21)	103.32 (17.40)	112.64 (26.04)	<0.001	<0.001
Triglyceride (mg/dL)	85.25 (39.43)	144.60 (97.82)	118.87 (66.40)	174.21 (120.36)	203.93 (119.85)	<0.001	<0.001
Total cholesterol (mg/dL)	190.23 (33.41)	199.30 (37.24)	196.04 (35.73)	203.22 (38.88)	206.20 (38.88)	<0.001	<0.001
HDL Cholesterol (mg/dL)	60.73 (12.44)	52.42 (10.49)	54.84 (10.77)	49.34 (9.19)	47.79 (8.54)	<0.001	<0.001
LDL Cholesterol (mg/dL)	111.52 (24.46)	122.01 (27.43)	118.19 (26.21)	126.98 (29.47)	128.65 (29.47)	<0.001	<0.001
AST (IU/L)	24.40 (9.71)	27.99 (13.79)	25.19 (11.98)	30.18 (13.79)	38.45 (18.25)	<0.001	<0.001
ALT (IU/L)	19.13 (9.47)	29.71 (23.49)	22.68 (15.75)	35.73 (27.04)	54.54 (28.89)	<0.001	<0.001
GGT (IU/L)	19.08 (15.64)	31.25 (29.55)	24.72 (22.61)	37.08 (32.83)	52.84 (41.04)	<0.001	<0.001
T. Bil (IU/L)	0.90 (0.36)	0.902 (0.345)	0.87 (0.33)	0.93 (0.31)	0.97 (0.45)	0.789	0.015
**Prevalence of sarcopenia, n (%)**							
ASM/BW*100	8 (1.3)	66 (8.8)	24 (5.5)	24 (9.7)	18 (28.1)	<0.001	<0.001
ASM/BMI	8 (1.3)	43 (5.8)	21 (4.8)	15 (6.0)	7 (10.9)	<0.001	<0.001
ASM/Ht^2^	12 (2.0)	6 (0.8)	5 (1.1)	1 (0.4)	0 (0)	0.055	0.284

**Note**: Data are presented as mean (SD) or number (percent); *p*-value *: Between NAFLD (−) and NAFLD (+) group. *t*-test or ANOVA were used for continuous variables, respectively. Fisher Exact test was used for categorical variables.

**Table 2:** Odds ratio (95% CI) of ASM and other variables for NAFLD at different model.

Odds Ratio of ASM	NAFLD (−)	NAFLD (+)	95% CI	*p*-Value
Unadjusted	1	1.12	1.093–1.147	<0.001
Model 1 (age)	1	1.143	1.114–1.172	<0.001
Model 2 (+height)	1	1.378	1.298–1.462	<0.001
Model 3 (+weight)	1	0.899	0.821–0.985	0.022
Model 4 (+metabolic parameters)	1	0.948	0.833–1.079	0.417

**Note:** Logistic regression was used to evaluate the OR between the NAFLD (−) and NAFLD (+) groups. Model 1: Adjusted for age; Model 2: Adjusted for age and height; Model 3: Adjusted for age, height, and weight; Model 4: Adjusted for age, height, weight, sex (M), AST, ALT, fasting glucose, Tg, and WC.

## Abbreviations

non-alcoholic fatty liver disease (NAFLD), fatty liver (FL), waist circumference (WC), height (Ht), body weight (BW), body mass index (BMI), appendicular skeletal muscle (ASM), total skeletal muscle (TSM), skeletal muscle index (SMI), weight-adjusted skeletal muscle index (wSMI), BMI-adjusted skeletal muscle index (bSMI), height-adjusted skeletal muscle index (hSMI), high-density lipoprotein (HDL), low-density lipoprotein (LDL), aspartate aminotransferase (AST), alanine aminotransferase (ALT), gammaglutamyltransferase (GGT), total bilirubin (T. Bil), direct bilirubin (D Bil), triglyceride (Tg).

## Appendix A

**Table A1:** Age-stratified body composition and prevalence of sarcopenia of study subjects according to hepatic steatosis status.

Total Subjects (N = 1343)	Normal	Mild FL	Moderate FL	Severe FL	*p* for Trend
**(a) Age (≥20 and <40), n = 394 (29.3%)**					
ASM (kg)	19.47 (5.19)	21.58 (4.61)	24.08 (2.90)	26.75 (3.15)	<0.001
TSM (kg)	25.92 (6.69)	28.54 (6.04)	31.14 (5.96)	35.80 (4.62)	<0.001
Fat (kg)	14.05 (4.43)	17.19 (5.87)	22.01 (6.53)	27.72 (7.96)	<0.001
wSMI	31.66 (3.65)	31.45 (3.83)	30.71 (2.49)	29.62 (2.38)	0.121
bSMI (m^2^)	0.9047 (0.1840)	0.9204 (0.1799)	0.9184 (0.1193)	0.9244 (0.1091)	0.870
hSMI (kg/m^2^)	6.77 (1.18)	7.36 (1.06)	8.06 (0.63)	8.57 (0.68)	<0.001
Sarcopenia n (%)					
by wSMI	1 (0.4)	3 (3.3)	4 (11.1)	4 (30.8)	<0.001
by bSMI	0 (0)	0 (0)	0 (0)	1 (7.7)	<0.001
by hSMI	3 (1.2)	0 (0)	0 (0)	0 (0)	0.64
**(b) Age (≥40 and <60), n = 758 (56.4%)**					
ASM (kg)	18.90 (4.52)	20.43 (4.43)	22.79 (3.63)	24.21 (4.10)	<0.001
TSM (kg)	25.04 (5.62)	27.23 (5.71)	30.20 (4.63)	32.09 (5.26)	<0.001
Fat (kg)	14.21 (4.23)	17.49 (5.01)	19.86 (5.20)	26.51 (8.22)	<0.001
wSMI	31.51 (4.04)	30.60 (3.55)	30.88 (3.00)	29.05 (3.07)	<0.001
bSMI (m^2^)	0.8709 (0.1832)	0.8556 (0.1666)	0.8925 (0.1343)	0.8478 (0.1415)	0.112
hSMI (kg/m^2^)	6.82 (1.08)	7.29 (1.01)	7.87 (0.83)	8.29 (0.85)	<0.001
Sarcopenia n (%)					
by wSMI	1 (0.4)	15 (5.4)	11 (6.4)	11 (26.8)	<0.001
by bSMI	2 (0.7)	14 (5.0)	10 (5.8)	4 (9.8)	0.004
by hSMI	6 (2.2)	3 (1.1)	0 (0)	0 (0)	0.165
**(c) Age (≥60), n = 191 (14.2%)**					
ASM (kg)	17.11 (4.21)	19.04 (4.29)	19.26 (4.46)	18.87 (3.28)	0.02
TSM (kg)	22.91 (5.23)	25.16 (5.56)	25.21 (6.80)	25.38 (4.19)	0.063
Fat (kg)	15.39 (4.55)	18.42 (4.98)	21.39 (5.22)	23.43 (6.93)	<0.001
wSMI	29.47 (4.03)	29.38 (3.62)	28.06 (4.04)	27.11 (3.33)	0.09
bSMI (m^2^)	0.7701 (0.1761)	0.7883 (0.1641)	0.7609 (0.1846)	0.7139 (0.1539)	0.598
hSMI (kg/m^2^)	6.54 (1.04)	7.09 (1.04)	7.12 (0.99)	7.20 (0.67)	0.003
Sarcopenia n (%)					
by wSMI	6 (8.0)	6 (9.2)	9 (22.0)	3 (30.0)	0.043
by bSMI	6 (8.0)	7 (10.8)	5 (12.2)	2 (20.0)	0.661
by hSMI	3 (4.0)	2 (3.1)	1 (2.4)	0 (0)	0.902

**Note**: Data are presented as mean (SD) or number (percent). ANOVA was used for continuous variables. Fisher Exact test were used for categorical variables.

**Table A2:** BMI-stratified prevalence of sarcopenia by two different adjustment method and in-concordance rate.

Total Subject (N = 1343)	Prevalence of Sarcopenia			In-Concordance Rate (%)
	By wSMI	By hSMI	By wSMI and hSMI	
Underweight (BMI < 18.5, n = 70)	0 (0)	7 (10.0)	0	10 (7/70)
Normal (BMI ≥ 18.5, <23, n = 538)	1 (0.2)	8 (1.5)	1	1.3 (7/538)
Overweight (BMI ≥ 23, <25, n = 355)	8 (2.3)	1 (0.3)	1	1.97 (7/355)
Obesity (BMI ≥ 25, n = 380)	65 (17.1)	2 (0.5)	2	16.57 (63/380)
*p* for trend	<0.001	<0.001	-	-

**Note**: Data are presented as number (percent). Pearson Chi-square test was used for calculating p for trend. In-concordance rate was calculated by dividing the difference between subjects with sarcopenia by wSMI and hSMI by the total number of subgroup in percent.
